# Assessment of the risk of burnout and its associated factors in healthcare professionals during the COVID-19 pandemic: A prospective cohort study

**DOI:** 10.3389/fpsyg.2023.1058417

**Published:** 2023-01-17

**Authors:** Rebeca R. C. Silva, Rodrigo C. Menezes, Stefania L. Garcia, Hugo N. Pustilnik, Isabella B. B. Ferreira, Kaique V. C. S. Aguiar, Nivaldo M. Filgueiras Filho, Mariana Araújo-Pereira, Bruno B. Andrade

**Affiliations:** ^1^Multinational Organization Network Sponsoring Translational and Epidemiological Research (MONSTER) Initiative, Salvador, Brazil; ^2^Escola Bahiana de Medicina e Saúde Pública (EBMSP), Salvador, Brazil; ^3^Instituto de Pesquisa Translacional e Clínica (IPCT), Faculdade de Tecnologia e Ciências, Salvador, Brazil; ^4^Laboratório de Inflamação e Biomarcadores, Instituto Gonçalo Moniz, Fundação Oswaldo Cruz, Salvador, Brazil; ^5^Faculdade de Medicina, Universidade Federal da Bahia (UFBA), Salvador, Brazil; ^6^Curso de Medicina, Universidade Salvador (UNIFACS), Salvador, Brazil; ^7^Unidade de Terapia Intensiva, Hospital EMEC, Feira de Santana, Brazil

**Keywords:** COVID-19, burnout syndrome, health professionals, ICU, beliefs, emergency

## Abstract

**Introduction:**

The COVID-19 pandemic resulted in tremendous physical and psychological pressure on healthcare professionals, especially on those working in intensive care units (ICUs) and Emergency Departments (EDs). The present study intended to characterize the profile of these professionals which is associated with burnout and determine the potential predictors of such condition.

**Methods:**

A Prospective cohort study was carried out in a tertiary hospital between March 2020 and March 2021, in Salvador, Brazil. A standardized and validated version of the Oldenburg Burnout inventory (OLBI) was applied to assess risk of burnout together with data forms designed to collect information on sociodemographic characteristics and religious beliefs. ICU and ED healthcare professionals were evaluated during off-hours at two distinct periods of the COVID-19 pandemic, in 2020 and in 2021. Differences in the results obtained from each study participant between the timepoints were compared. A binary logistic regression analysis was performed to identify the predictors of burnout development independent of other confounding factors.

**Results:**

Seventy-seven healthcare professionals with a median age of 33 (interquartile range [IQR]: 31–37.5) years and predominantly female (72.7%; *n* = 56) were enrolled. There were 62 professionals at risk of developing burnout through the OLBI. Those had a median age of 33 (IQR: 31–37) and female predominance (71%, *n* = 44). Disengagement and burnout were the only features which frequencies significantly changed over time, with increasing detection at the latest timepoint. Alcohol consumption was found to be an important risk factor for burnout development [adjusted odds ratio (aOR): 10.8 (95% CI: 1.8–64.2)]. Importantly, working in the ICU [aOR: 0.04 (95%CI: 0.01–0.32)] and the habit of praying daily [aOR: 0.07 (95%CI: 0.01–0.41)] were characteristics linked to reduced odds of burnout.

**Discussion:**

Disengagement substantially increased during the COVID-19 pandemic in healthcare professionals. Alcohol consumption favors the onset of burnout whereas habit of praying daily and working in the ICU are protective against such outcome. Institutional policies aimed at minimizing etilism may positively impact mental health of these professionals.

## 1. Introduction

Healthcare professionals who have worked during the COVID-19 pandemic suffered a high level of stress and anxiety, especially those in Intensive Care Units (ICU) and Emergencies (Dos Santos Barros et al., 2016). Studies have shown that these specialists have become a population that is particularly vulnerable to excessive levels of fear, fatigue and irritability due to their social and professional responsibility, in addition to the strenuous workday, due to the outrageous rapid increase in severe COVID-19 cases, overloading the healthcare services (de Oliveira et al., 2020; Di Tella et al., 2020; Dosil et al., 2020; Romero et al., 2020; Rossi et al., 2020; Rumeysa et al., 2020; Arpacioglu et al., 2021; Cipolotti et al., 2021).

Although “staff burn-out” was designated by Freudenberg in the 70’s as an experience of disappointment, physical and emotional exhaustion and loss of interest in work, the discussion about Burnout has substantially expanded over the recent years and robustly intensified during the COVID-19 pandemic. Currently, researchers define this condition as a psychological stress related to work and its main manifestations would be exhaustion and disengagement, but above all, exhaustion (Demerouti et al., 2001; Bakker et al., 2004; Demerouti, 2008; da Silva Schuster and da Veiga Dias, 2018). Previous studies have underscored that, as in other epidemics, the impact on mental health is strongly related to the greater number of individuals affected by the infectious contagion itself (Reardon, 2001; Ornell et al., 2020). Not only, factors such as long working hours, high workloads, lack of resources, and inadequate staffing have been identified as potential stressors, with the responsibilities of health professionals potentially exacerbating this phenomenon (West et al., 2016).

Furthermore, the insecurity, fear and sadness that permeate the scenario observed during the COVID-19 pandemic have revealed a chain of concepts related to sustaining what is in fact essential to life. Since spirituality has the potential to promote the idea of protection and humanization, by manifesting the character of hope, mastery of resilience and promotion of well-being, some healthcare professionals were able to exercise their belief as a coping strategy (Tavares, 2020). The primary goal of this study was to determine which characteristics can predict the development of burnout in healthcare professionals. Additionally, this study sought to measure the relationship of faith as a coping strategy trough a questionnaire of religious beliefs.

## 2. Methods

### 2.1. Study design and participants

**Figure 1:**
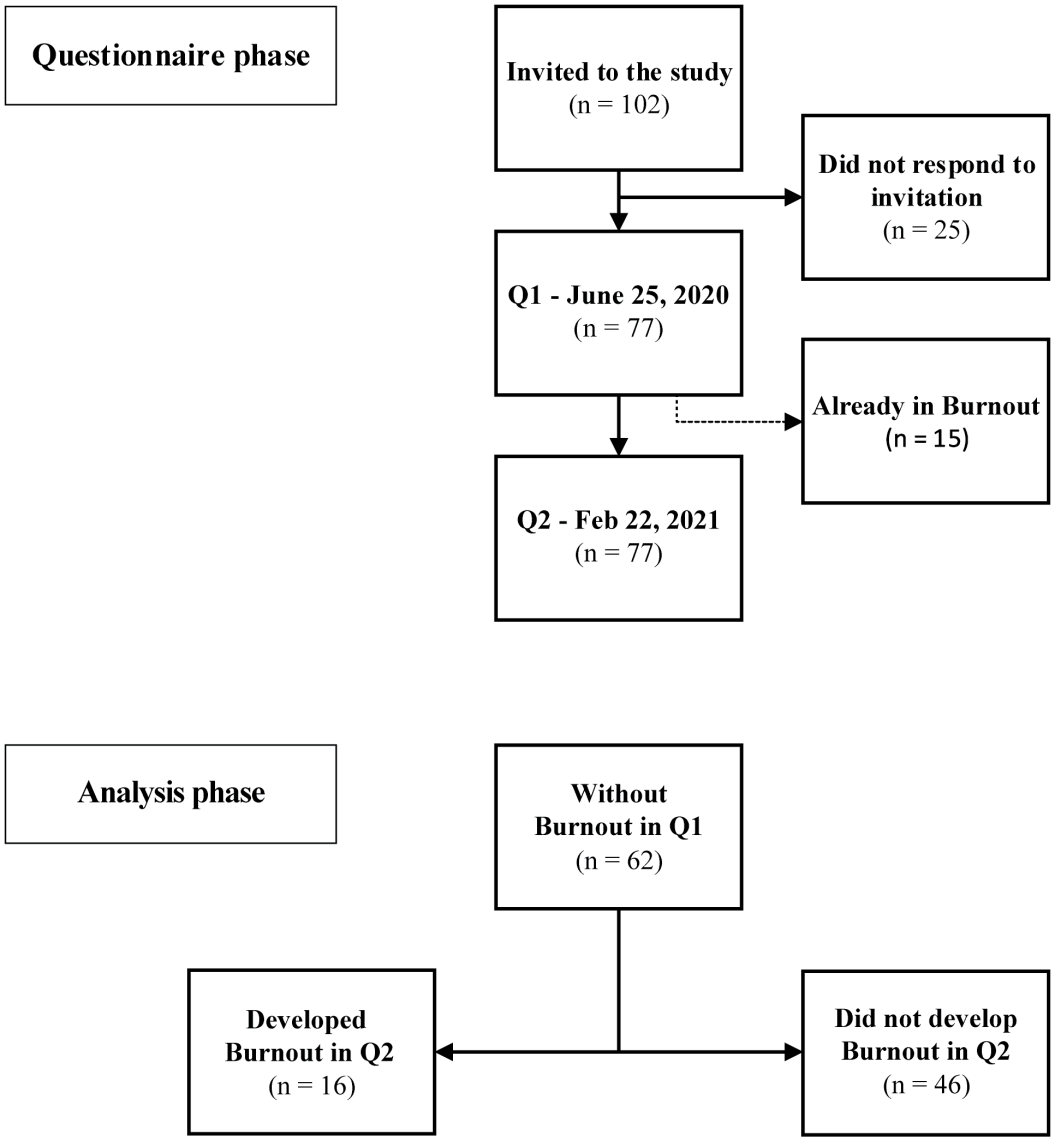
Study flowchart. The questionnaires phase demonstrates the time-points in which they were applied. There was no loss of follow-up between study periods. The analytical phase included only health professionals who did not have burnout in Q1.

Prospective, descriptive, and analytical cohort, from June 2020 to March 2021, which included all health professionals from the emergency department and ICU, that provided consent, of a large COVID-19 referral hospital located in the state of Bahia, Brazil. Of 102 health professionals, 77 agreed to participate in this work (Figure 1).

### 2.2. Procedures

A self-administered online data questionnaire was applied at two different timepoints of the COVID-19 pandemic during commencement of off-work hours, first on June 25, 2020 (Q1), then on February 22, 2021 (Q2) (Figure 1). The first application of the questionnaire intends to identify those who are already in burnout and, therefore, are not able to develop Burnout developing the syndrome in Q2, in addition to discerning the baseline characteristics of the studied population. The second application aims to assess changes in baseline characteristics and to identify those who developed the syndrome during the study period. The information acquired in Q1 was used in a logistic model to predict the development of burnout in Q2.

Both questionnaires contained three sections: the first obtained socio-demographic data (age, gender, ethnicity, weight, occupation, financial satisfaction, chronic illnesses, physical activity, drug consumption, dissatisfaction with eating habits, psychological support and isolation due to COVID-19); the second section contained the questions that made up OLBI; the third section contained the questions that made up Beliefs. Details of these last two are described below. A copy of the questionnaire translated to English is shown in Supplementary File S1.

#### 2.2.1. Oldenburg burnout inventory

A validated Portuguese translation of the standardized OLBI was used to assess burnout risk (da Silva Schuster and da Veiga Dias, 2018). It consists in a self-report four-point Likert scale with eight questions for each dimension: disengagement and exhaustion (Demerouti et al., 2001). In such questionnaire, disengagement refers to distancing from work in terms of both object and content, and the development of cynical and negative attitudes (Bakker et al., 2004). Exhaustion is the feeling of physical fatigue, the need to rest, and feelings of overtaxing and emptiness in relation to work (Demerouti, 2008). As used in recent studies, mean scores ≥ 2.25 in exhaustion were considered as high exhaustion, whereas scores ≥ 2.1 on disengagement were considered as high disengagement. The presence of the two high scores indicated Burnout.

#### 2.2.2. Beliefs

Furthermore, the long form of the standardized Beliefs questionnaire was also applied. This questionnaire was published in a report of the Fetzer Institute and the National Institute on Aging Working Group in a compilation of multidimensional measurement tools of religiousness and spirituality for use in health research. It consists in a self-report, not validated, five-point rating scale with seven questions that assess the degree of an individual’s beliefs. Its sum was used to estimate the degree of personal faith. The cognitive dimension of belief is central to religiosity and, despite differences from religion to religion, there is in all of them a search for the meaning of suffering and death, bringing comfort and serving as a coping mechanism (Fetzer Institute, and National Institute on Aging Working Group, 1999).

### 2.3. Statistical analysis

Median values with interquartile ranges (IQR) were used as measures of central tendency and dispersion, respectively. The Mann–Whitney *U* test (for two unmatched groups) and the Wilcoxon matched pair test (for two matched groups) were used to compare continuous variables. The categorical variables were compared using the Fischer’s exact test (in 2 × 2 tables) and were presented as number and frequency (%). A binary regression analysis, backwards stepwise method, was used to identify factors independently associated with burnout development. All variables that presented value of *p* < 0.1 in the univariable analysis were included in the final adjusted model. Differences with value of *p*s < 0.05 were considered statistically significant. Statistical analyzes were performed in R (version 4.1.1).

## 3. Results

### 3.1. Characteristics of the study participants at baseline

**Figure 2:**
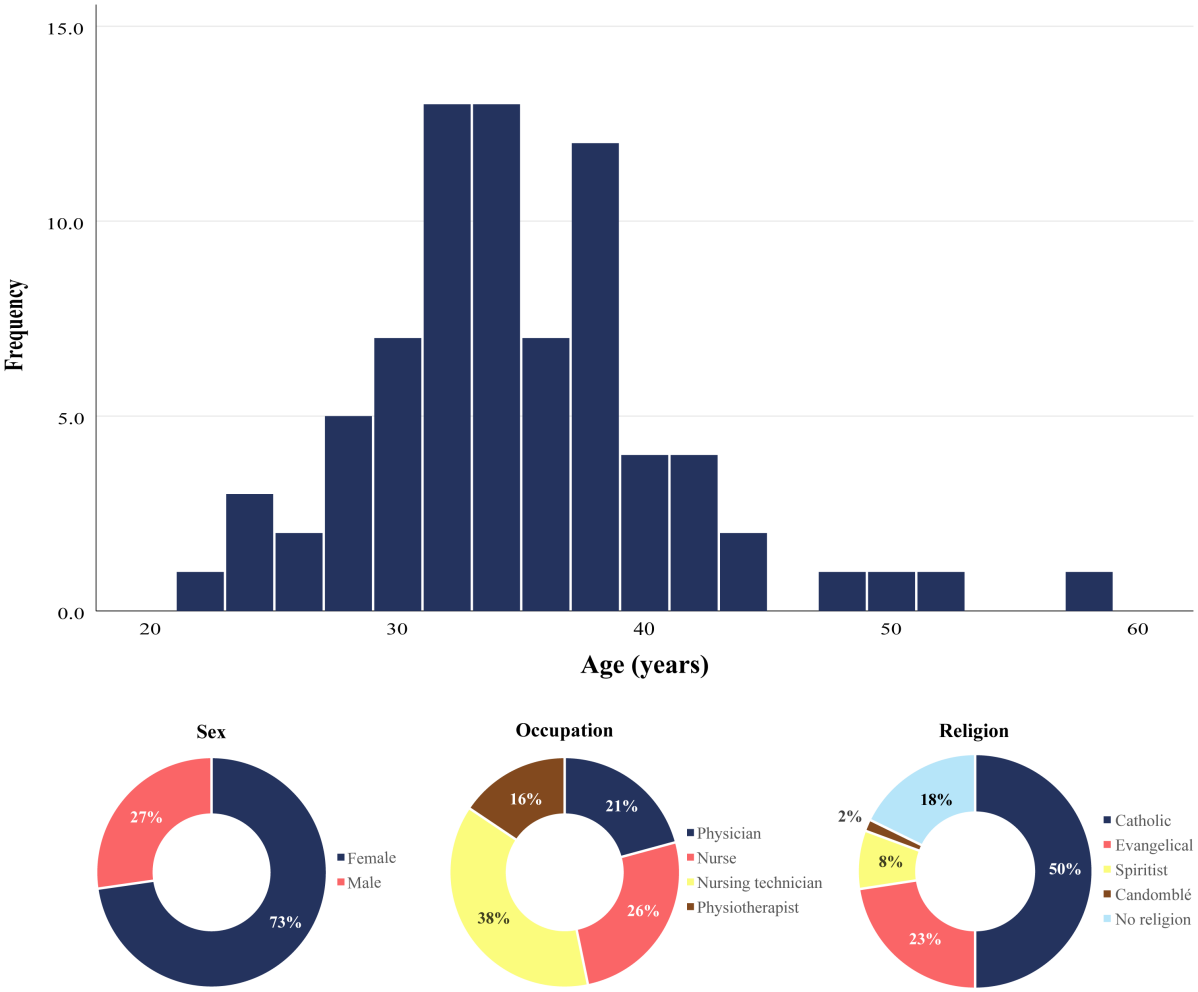
Population Characteristics. Upper panel: histogram depicting age distribution of the study participants at the baseline. Lower panel: Distribution of sex, occupation and religion types among the study participants. Data are frequencies of a total of 77 individuals who enrolled the study.

The median age of the population was 33 (IQR: 31–37) years, there was a predominance of women (72.7%; *n* = 56) and the most prevalent self-reported skin color was brown (61%; *n* = 47; Figure 2). There were 16 (20.8%) physicians, 20 (26.0%) nurses, 29 (37.7%) nursing technicians and 12 (15.6%) physiotherapists, 54 of whom worked in the ICU, 14 worked in the emergency department and 8 worked in both sectors. Also, 50 (64.9%) participants reported a monthly income lower than 5 minimum wages, 57 (74%) did less than 2 h of physical activity, 41 (53.2%) considered its nutrition inadequate, and 1 (1.3%) smoked. At baseline, 15 (19.5%) professionals were already in Burnout.

### 3.2. Change in population profile over time during the COVID-19 pandemic

**Figure 3:**
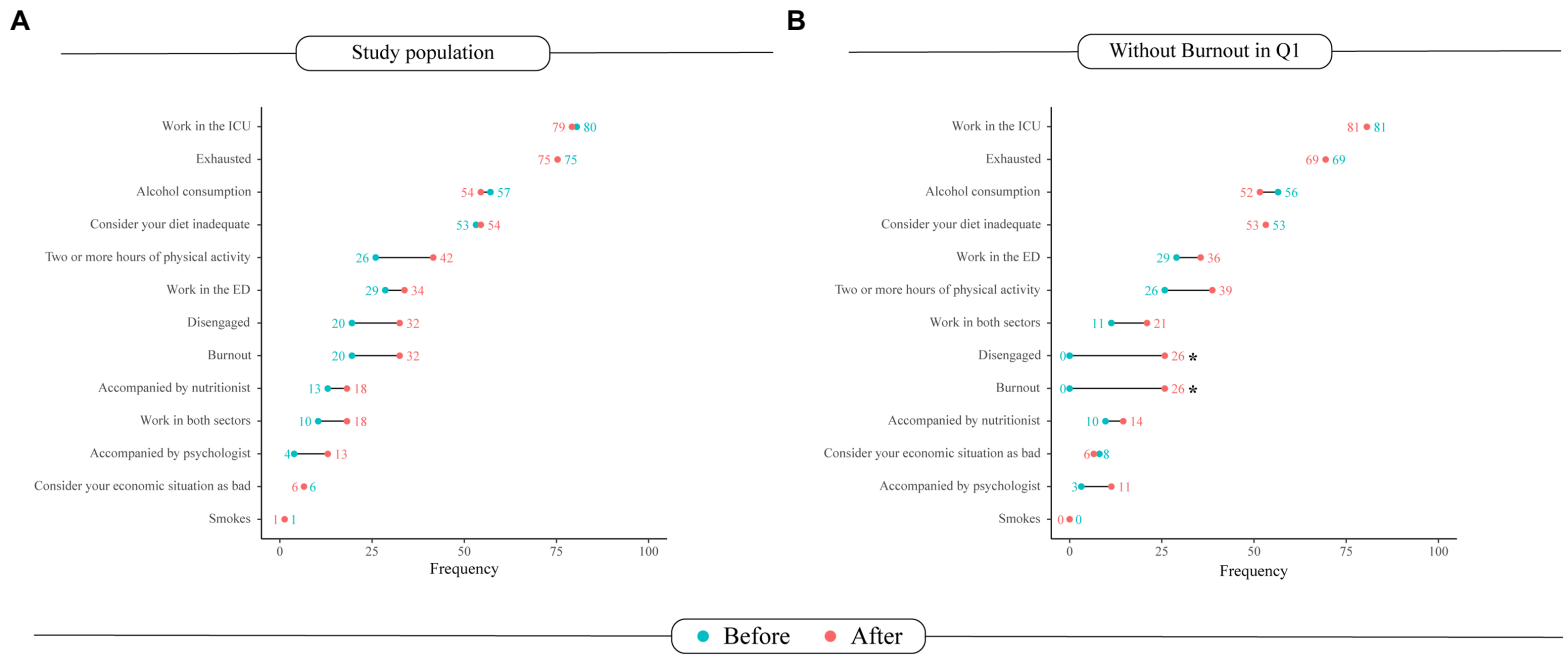
Changes in the Burnout questionnaire in the **(A)** overall study population and in the **(B)** participants who have not developed burnout. In blue, the frequencies of the characteristics in the first questionnaire are represented as % of the study participants. In red, frequencies obtained in the second questionnaire. Asterisks indicate statistically significant differences (value of *p* < 0.05). Intensive Care Unit (ICU), Emergency Department (ED).

The Figure 3 summarizes the changes in the frequency of the characteristics evaluated in this study between the two timepoints. No variation was statistically significant. The most substantial difference was observed in the practice of physical activity, which showed a relative increase of 76.9% (from 26 to 46%, value of *p* = 0.060). Furthermore, the proportion of exhausted professionals remained similar between the timepoints, the increase in disengaged participants (from 20 to 32%, value of *p* = 0.097) being responsible for an increase of the same proportion of individuals in burnout. Accordingly, the rate of participants undergoing nutritional and psychological assistance rose slightly, respectively from 13 to 18.2% (value of *p* = 0.506) and 3.9 to 13% (value of *p* = 0.079).

### 3.3. Burnout development assessment

**Figure 4:**
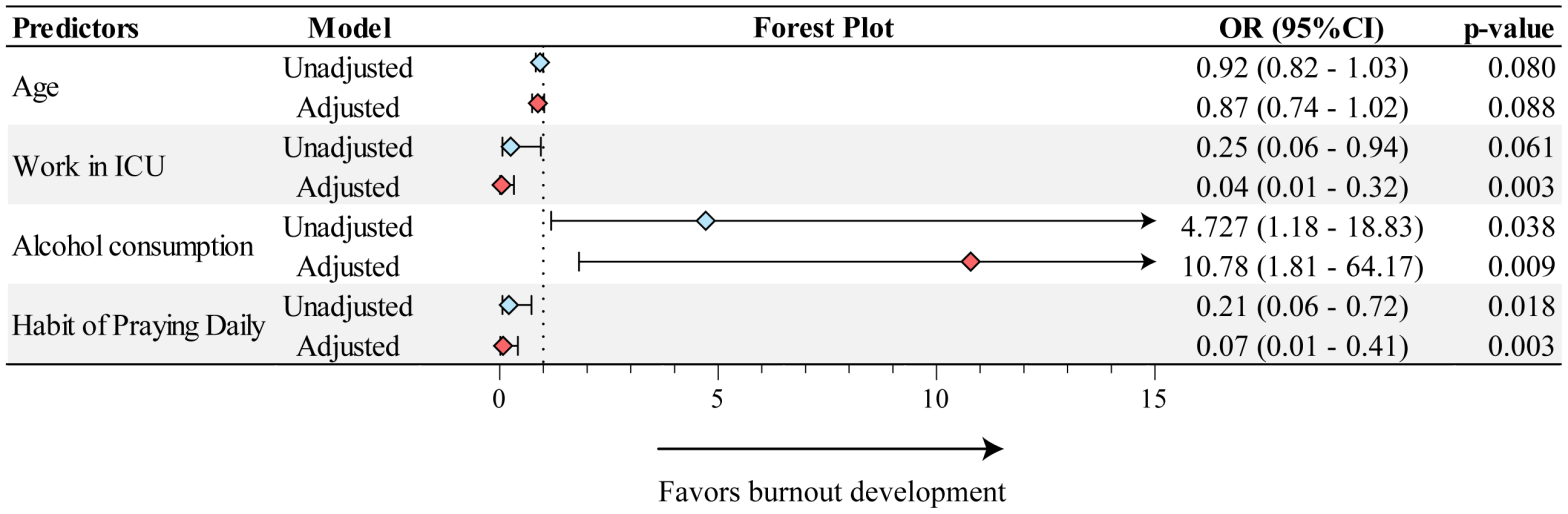
Adjusted and unadjusted binary regression model for Burnout development. The model presents only the variables which passed on the final step of the regression analysis. Multivariable regression was adjusted for differences in values characteristics with statistical relevance (value of *p* < 0.1). Odds Ratio (OR); Intensive Care Unit (ICU).

This stage of the analysis included only professionals who, in Q1, had no Burnout (*n* = 62). Those had a median age of 33 (IQR: 31–37) and a female predominance (71%, *n* = 44). The only variables that showed a significant change in frequency throughout the study were disengagement and burnout (Figure 3B). Regarding burnout development, there were no significant differences in age, sex, work department and self-reported skin color (Table 1). The logistic regression analysis model revealed two predictors of burnout that were independent of other confounding factors. The predictors were: working in the ICU (adjusted OR: 0.04; 95%CI: 0.01–0.32) and alcohol consumption (adjusted OR: 10.8; 95%CI: 1.8–64.2). Importantly, the habit of praying daily was shown to be a protective feature minimizing the odds of burnout in such adjusted analysis (adjusted OR: 0.07; 95%CI: 0.01–0.41; Figure 4).

**Table 1:** Comparison according to the development of burnout.

Characteristic	General population (*n* = 62)	No burnout (*n* = 46)	Burnout (*n* = 16)	value of *p*
Age years	33 (31–37.5)	34 (31–38)	32.5 (29.5–34)	0.080
Sex female	44 (71%)	32 (69.6%)	12 (75%)	0.760
Occupation				
Doctor	15 (24.2%)	12 (26.1%)	3 (18.8%)	0.739
Nurse	15 (24.2%)	7 (15.2%)	8 (50%)	**0.014**
Nursing technician	22 (35.5%)	20 (43.5%)	2 (12.5%)	**0.034**
Physiotherapist	10 (16.1%)	7 (15.2%)	3 (18.8%)	0.709
Work in ICU	43 (80.6%)	34 (73.9%)	9 (56.3%)	0.061
Work in ED	11 (29.0%)	6 (13.0%)	5 (31.3%)	0.523
Have children	32 (51.6%)	26 (56.5%)	6 (37.5%)	0.250
Skin color				0.109
White	9 (14.5%)	5 (10.9%)	4 (25%)	
Brown	41 (66.1%)	29 (63%)	12 (75%)	
Black	11 (17.7%)	11 (23.9%)	0 (0%)	
Yellow	1 (1.6%)	1 (2.2%)	0 (0%)	
Chronic diseases	9 (14.5%)	6 (13%)	3 (18.8%)	0.683
Consider his economic situation as bad	5 (8.1%)	4 (8.7%)	1 (6.3%)	1.000
Monthly income <5 minimum wages	40 (64.5%)	31 (67.4%)	9 (56.3%)	0.546
Less than 2 h of physical activity	46 (74.2%)	36 (78.3%)	10 (62.5%)	0.319
Consider your diet inadequate	33 (53.2%)	24 (52.2%)	9 (56.3%)	1.000
Accompanied by nutritionist	6 (9.7%)	3 (6.5%)	3 (18.8%)	0.172
Alcohol consumption	35 (56.5%)	22 (47.8%)	13 (81.3%)	**0.038**
Accompanied by psychologist	2 (3.2%)	1 (2.2%)	1 (6.3%)	0.453
Performed home isolation	12 (19.4%)	9 (19.6%)	3 (18.8%)	1.000
Religion				0.412
Catholic	31 (50%)	24 (52.2%)	7 (43.8%)	
Evangelical	14 (22.6%)	10 (21.7%)	4 (25%)	
Spiritism	5 (8.1%)	2 (4.3%)	3 (18.8%)	
Candomblé*	1 (1.6%)	1 (2.2%)	0 (0%)	
No religion	11 (17.7%)	9 (19.6%)	2 (12.5%)	
Visits holy sites every week	17 (27.4%)	16 (34.8%)	1 (6.3%)	**0.048**
Habit of praying daily	46 (74.2%)	38 (82.6%)	8 (50%)	**0.018**
BELIEFS score	11 (10–13)	11.5 (10–13)	11 (9.5–13)	0.626

Continuous values are represented as medians and (interquartile ranges), categorical values are represented as frequency (%). In bold, *p*-values < 0.05, representing the comparison between burnout and no burnout. Intensive care unit (ICU). Emergency department (ED). *Camdomblé is an African diasporic religion that developed in Brazil during the 19th century.

## 4. Discussion

This study evaluated the occurrence of burnout in a population under the prolonged effect of a stressful event, which was the COVID-19 pandemic, and identified the predictors for the development of such syndrome, in a country heavily affected by SARS-CoV-2 plague. The study participants represent the population of healthcare professionals who worked on the frontlines in the fight against the pandemic. One important contribution of this study was the observation that Burnout has no predilection for age, sex, race/skin color and work department (either ED or ICU). However, there appeared to be an association regarding the area of activity, in which nurses and nursing technicians were differentially predisposed to develop burnout, in line with another study carried out with Swedish health professionals, including nurses, midwives and biomedical technicians who developed burnout (Peterson et al., 2008). Of note, these relationships were not sustained after adjustment of other confounding factors in the present study, indicating that additional investigations are required to clarify whether area of activity of healthcare professionals is indeed an independent contributing factor for burnout development in other settings than those depicted here.

Despite the challenges that arose in the daily lives the professionals, such as the tiring and indispensable requirement for the use and removal of all Personal Protective Equipment (PPE) necessary to guarantee collective protection (Cabarkapa et al., 2020), the strenuous working hours and inadequate working conditions, it should be noted that those professionals were already exhausted before the pandemic and remained exhausted during the period we applied the second questionnaire. In this setting, the most significant change in profile was driven by “disengagement.” According to a study of Romanian psychiatric residents, all participants who experienced high burnout (*n* = 26) were satisfied with their salary, but dissatisfied with the resources available for patient care (Tipa et al., 2019). These factors are capable of intensifying negative symptoms, such as feelings of vulnerability, powerlessness and fear, resulting in an exacerbated effort in the workplace with limited opportunities for recovery (Bai et al., 2004; Talaee et al., 2022). These conditions were more common in healthcare professionals who had difficulty sleeping, difficulty concentrating, feelings of indifference and of anxiety, in agreement with another study of Swedish healthcare professionals in which the data revealed self-reported depression, anxiety, sleep, memory impairment, neck and back pain (Peterson et al., 2008). A study of 227 physicians reported some ways to reduce anxiety and burnout rates, through interventions that taught them about the psychology of burnout, stress, coping with patient death and suffering management, as well as providing information about prevalence rates among physicians. The results were based on a before-and-after analysis of symptoms, producing a significant decrease in physicians’ levels of burnout and anxiety. From this, it can be inferred that despite the fact that burnout has several triggers, anxiety probably is one of the main intrinsic and potentiating factors of the syndrome, considering that it was the only factor that had an important decrease concomitant with exhaustion (Medisauskaite and Kamau, 2019).

To the detriment of the context of the COVID-19 pandemic, there is a need for these professionals to have coping strategies to attenuate negative symptoms. Therefore, our study had a secondary aim to sought to measure the relationship of faith as a coping strategy through a non-validated questionnaire of religious beliefs and habits. The habit of praying every day was a significant protective factor for burnout, and this may occur through the perception of divine protection, as mentioned by Tavares Quelho, while discussing spirituality as a dimension of protection and humanization, in addition to bringing the character of hope (Tavares, 2020). A different study with healthcare professionals in Hong Kong demonstrated in a regression analysis that daily spiritual practice acts as a protective factor not only against burnout, but also against depression and anxiety (Shaniuk, 2020).

Some coping strategies, such as substance abuse, may have positive short-term effects, but can have negative long-term repercussions (Costa and Moss, 2018). Our findings reveal that a significant portion of the study population consumed alcohol, and this feature was an independent risk factor for the development of burnout. This observation reinforces the hypothesis that alcohol abuse is associated with higher levels of burnout. Likewise, the Swedish study of healthcare professionals found that alcohol consumption was indeed the factor that most clearly differentiated the exhausted/disengaged group from the non-exhausted group (Peterson et al., 2008; Galaiya et al., 2020).

Importantly, the present study has some limitations. This was a single-center study that may suffer from unmeasured effects. Due to the small sample size, a more detailed analysis of habits was not carried out, such as the quantification of alcohol consumption, the modality of physical activity or the assessment of personality traits. The latter can influence the associations described in this study. People with higher levels of extroversion and openness are more likely to drink alcohol, and individuals with higher levels of neuroticism may have difficulty managing their emotions and may turn to alcohol as a coping mechanism. Not only that, higher levels of neuroticism can turn a person away from religious beliefs (Khoynezhad et al., 2012; Lui et al., 2022). Regarding the interpretation of the spiritual influence and religious beliefs on the professional’s mental health status, even with the Beliefs scale, faith is not objectively measurable, and there is no gold standard for the analysis of this variable. As a weakness, religious beliefs may lead to a false sense of security and delay professional assistance (Koenig, 2012). Regardless, the results presented here are relevant an contribute to the knowledge in the field as they demonstrate a solid interaction between specific characteristics of healthcare professionals and occurrence of burnout in a population that dramatically suffered with the COVID-19 pandemic in one of the countries which had the highest disease burden in the world.

## 5. Conclusion

Among Brazilian healthcare professionals from ED and ICU from a hospital directly affected by COVID-19 pandemic, alcohol use was the strongest factor predisposing to increasing risk of burnout whereas the habit of praying daily and working in the ICU were protective against such syndrome. These findings may guide decision-making strategies to implement institutional measures to reduce risk of burnout in extreme conditions such as the catastrophic scenario caused by the SARS-CoV-2 virus.

## Data availability statement

The original contributions presented in the study are included in the article/Supplementary material, further inquiries can be directed to the corresponding author.

## Ethics statement

The studies involving human participants were reviewed and approved by Research Ethics Committee of Federal University of Bahia. The patients/participants provided their written informed consent to participate in this study.

## Author contributions

RS, RM, IF, SG, HP, NF, and BA: conceptualization, design of study, and manuscript draft. RS, RM, IF, and KA: investigation and visualization. RS, RM, NF, and KA: data acquisition. RS, RM, IF, MA-P, SG, HP, and BA: data analysis and interpretation. RS, RM, IF, SG, HP, and BA: supervision, critical revision, editing and final approval of the manuscript. All authors read and approved the final manuscript.

## Funding

This study was supported by the Intramural Research Program of the Oswaldo Cruz Foundation, Brazil.

## Conflict of interest

The authors declare that the research was conducted in the absence of any commercial or financial relationships that could be construed as a potential conflict of interest.

## Publisher’s note

All claims expressed in this article are solely those of the authors and do not necessarily represent those of their affiliated organizations, or those of the publisher, the editors and the reviewers. Any product that may be evaluated in this article, or claim that may be made by its manufacturer, is not guaranteed or endorsed by the publisher.
