# Supplementary material for: Assessment of the risk of burnout and its associated factors in healthcare professionals during the COVID-19 pandemic: A prospective cohort study
Source: Front Psychol. 2023 Jan 17;14:1058417. doi: 10.3389/fpsyg.2023.1058417 (PMC9887157; doi:10.3389/fpsyg.2023.1058417)
Supplement: Supplementary file 1 [file Data_Sheet_1.PDF]

Questionnaire

*Required

Email: ________________________

Telephone: ________________________

Sociodemographic Questionnaire

Age: ________________________

Weight: ________________________

You currently work:

Tick all that apply.

- [ ] Emergency
- [ ] ICU

Did you need to isolate yourself from your children or partner during the pandemic?

Tick all that apply.

- [ ] Yes
- [ ] No

Do you have any chronic diseases? *

Mark only one oval.

- ( ) Yes
- ( ) No

How do you rate your economic situation today? *

Mark only one oval.

- Good
- Bad

Currently, your monthly income is:

Mark only one oval.

- Less than 5 minimum wages
- More than 5 minimum wages

Do you perform two or more hours of physical activities regularly?

Mark only one oval.

- Yes
- No

Currently, how do you consider your diet? *

Mark only one oval.

- I do nutritional monitoring and follow what is recommended to me.
- I do nutritional accompaniment, but I don't follow to the letter.
- I don't do nutritional accompaniment, but I think it's appropriate.
- I don't do nutritional accompaniment and I think I could improve.

Smoking? *

Mark only one oval.

- Yes
- No

Alcohol consumption *

Mark only one oval.

- Yes
- No

Do you consider yourself dependent on alcohol? *

Mark only one oval.

- Yes
- No

Other substances? *

Mark only one oval.

- Yes
- No

If so, which ones?

Are you accompanied by a psychologist?

Mark only one oval.

- Yes
- No

Were you removed from work on suspicion of COVID-19? *

Mark only one oval.

- Yes
- No

Did you develop any sleep disorders during the pandemic? *

Tick all that apply.

- [ ] Yes
- [ ] No

When you wake up, you feel: *

Mark only one oval.

- Refreshed
- Tired

Do you have frequent nightmares?

Mark only one oval.

- Yes
- No

Do you feel excessive fear? *

Mark only one oval.

- Yes
- No

Do you notice any difficulty concentrating? *

Mark only one oval.

- Yes
- No

Do you have episodes of forgetfulness? *

Mark only one oval.

- Yes
- No

Do you have a feeling of indifference to others? *

Mark only one oval.

- Yes
- No

Do you have any persistent negative emotional state? (e.g. stress, discouragement, sadness, anxiety, dread, anger, guilt, shame, etc.)

Mark only one oval.

- Yes
- No

Which? ______________________

Section 2

Below you will find several statements with which you can agree or disagree.

I always find interesting new aspects in my work. *

Mark only one oval.

- I totally agree
- I partially agree
- I partially disagree
- I totally disagree

There are days when I feel tired before I get to work. *

Mark only one oval.

- I totally agree
- I partially agree
- I partially disagree
- I totally disagree

I'm talking more and more about my work in a negative way. *

Mark only one oval.

- I totally agree
- I partially agree
- I partially disagree
- I totally disagree

After work I usually need more time than in the past to relax and feel better.

Mark only one oval.

- I totally agree
- I partially agree
- I partially disagree
- I totally disagree

I can tolerate the pressure of my work very well. *

Mark only one oval.

- I totally agree
- I partially agree
- I partially disagree
- I totally disagree

Lately, I tend to think less while I work and do it almost mechanically.

Mark only one oval.

- I totally agree
- I partially agree
- I partially disagree
- I totally disagree

I consider my work a positive challenge. *

Mark only one oval.

- I totally agree
- I partially agree
- I partially disagree
- I totally disagree

During my work, I often feel emotionally exhausted. *

Mark only one oval.

- I totally agree
- I partially agree
- I partially disagree
- I totally disagree

Over time, you can feel disconnected from this type of work.

Mark only one oval.

- I totally agree
- I partially agree
- I partially disagree
- I strongly disagree

After working, I have enough energy for my leisure activities. *

Mark only one oval.

- I totally agree
- I partially agree
- I partially disagree
- I totally disagree

Sometimes I feel disgusted by my work tasks. *

Mark only one oval.

- I totally agree
- I partially agree
- I partially disagree
- I totally disagree

After my work, I usually feel exhausted and tired. *

Mark only one oval.

- I totally agree
- I partially agree
- I partially disagree
- I totally disagree

This is the only job I can imagine doing. *

Mark only one oval.

- I totally agree
- I partially agree
- I partially disagree
- I totally disagree

Generally, I can manage the amount of my work well. *

Mark only one oval.

- I totally agree
- I partially agree
- I partially disagree
- I totally disagree

I feel increasingly involved in my work. *

Mark only one oval.

- I totally agree
- I partially agree
- I partially disagree
- I totally disagree

When I work, I usually feel energized *

Mark only one oval.

- I totally agree
- I partially agree
- I partially disagree
- I totally disagree

I feel like I don't rest and i'm going back to work even more tired *

Mark only one oval.

- I totally agree
- I partially agree
- I partially disagree
- I totally disagree

How much is spirituality a source of strength and comfort for you? *

Mark only one oval.

- It isn’t
- A little
- Very much

Do you believe there's an afterlife? *

Mark only one oval.

- Yes
- No
- I don’t know

Divine goodness is greater than we can imagine. *

Mark only one oval.

- I strongly agree
- I partially agree
- I don't know
- I partially disagree
- I strongly disagree

Even though things go wrong, the world is still moved by love. *

Mark only one oval.

- I strongly agree
- I partially agree
- No sei
- I partially disagree
- I strongly disagree

When I come up with a tragic event, I try to remember that God still loves me and that there is hope for the future.

Mark only one oval.

- I strongly agree
- I partially agree
- I don't know
- I partially disagree
- I strongly disagree

I feel it's important to my kids that they believe in something. *

Mark only one oval.

- I strongly agree
- I partially agree
- No sei
- I partially disagree
- I strongly disagree

I believe that everything happens for a reason *

Mark only one oval.

- I strongly agree
- I partially agree
- No sei
- I partially disagree
- I strongly disagree

My faith has kept me on my feet during the pandemic. *

Mark only one oval.

- I totally agree
- I partially agree
- No sei
- I partially disagree
- I totally disagree

Do you have any religion? *

Mark only one oval.

- Yes
- No

If yes, which: _______

Do you attend churches/temples/*terreiros* every week?

Mark only one oval.

- Yes
- No

Do you have a habit of praying every day? *

Mark only one oval.

- Yes
- No
